# Attention-Based Multi-NMF Deep Neural Network with Multimodality Data for Breast Cancer Prognosis Model

**DOI:** 10.1155/2019/9523719

**Published:** 2019-05-13

**Authors:** Hongling Chen, Mingyan Gao, Ying Zhang, Wenbin Liang, Xianchun Zou

**Affiliations:** ^1^College of Computer and Information Science, Southwest University, Chongqing 400715, China; ^2^Key Laboratory of Luminescent and Real-Time Analytical Chemistry (Southwest University), Ministry of Education, College of Chemistry and Chemical Engineering, Southwest University, Chongqing 400715, China

## Abstract

Today, it has become a hot issue in cancer research to make precise prognostic prediction for breast cancer patients, which can not only effectively avoid overtreatment and medical resources waste, but also provide scientific basis to help medical staff and patients family members to make right medical decisions. As well known, cancer is a partly inherited disease with various important biological markers, especially the gene expression profile data and clinical data. Therefore, the accuracy of prediction model can be improved by integrating gene expression profile data and clinical data. In this paper, we proposed an end-to-end model, Attention-based Multi-NMF DNN (AMND), which combines clinical data and gene expression data extracted by Multiple Nonnegative Matrix Factorization algorithms (Multi-NMF) for the prognostic prediction of breast cancer. The innovation of this method is highlighted through using clinical data and combining multiple feature selection methods with the help of Attention mechanism. The results of comprehensive performance evaluation show that the proposed model reports better predictive performances than either models only using data of single modality, e.g., gene or clinical, or models based on any single NMF improved methods which only use one of the NMF algorithms to extract features. The performance of our model is competitive or even better than other previously reported models. Meanwhile, AMND can be extended to the survival prediction of other cancer diseases, providing a new strategy for breast cancer prognostic prediction.

## 1. Introduction

Nowadays, cancer has become the leading cause of morbidity and mortality worldwide, in which breast cancer is one of the most common malignant tumors, especially among women [1–9]. According to the statistics, around the world, an estimated 1.2 million women are diagnosed with breast cancer as well as around 50 million women died of breast cancer each year. Hence, it is urgent to develop efficient computational methods to predict the survival time of breast cancer patients more precisely and promote the development of personalized treatment and management. At the same time, accurate prognostic prediction for breast cancer is of vital importance for the clinical decision of early breast cancer patients in adjuvant therapy. It is never easy to make decisions about patient treatment because it depends on a variety of clinical characteristics, genomic factors, tumor pathology, and cell classification [10, 11], in which clinical data and gene expression profile data are the most typical data for cancer prognosis prediction. Accordingly, more accurate prediction of cancer prognosis can not only help breast cancer patients understand their life expectancy, but also help clinicians make informed decisions and further guide the follow-up treatment. Thus, it would ultimately contribute to reducing overall mortality rate of breast cancer and further improving the quality of life of breast cancer patients.

During the past decades, gene expression profiling has become a powerful instrument for studying the biology of breast cancer. With these techniques, many prognostic features in gene expression can predict breast cancer recurrence risk [12]. Van de Vijver et al. [13] took multivariate analysis method to find out 70 genetic makers related to survival time of breast cancer from the gene expression data of 98 breast cancer patients. Their work indicates that the genetic markers play a significant role in the prediction of breast cancer survival time, but this method only requires simple genetic markers screening methods such as multivariate analysis, which still remains flawed. Gene expression data is high-dimensional and contains a large number of genes, resulting in a limited efficiency for these gene expression-based technologies. Therefore, in order to efficiently extract characteristic genes in high-dimensional data, Xu et al. [14] proposed a feature selection method based on support vector machine (SVM) for the selection of key features in data. This method uses two-step feature selection algorithm to process high-dimensional feature set in order to select characteristic that can help prediction. Their results show that the feature selection method based on machine learning is superior to the traditional artificial one. However, the above methods were only performed around single-modal data of gene expression, and important features related to breast cancer prognosis in other omics data (such as clinical data) were not considered. To take into account multimodality data and improve the accuracy of breast cancer prognosis predictions, Gevaert et al. [15] proposed a prediction algorithm based on probability graph, which fully integrated two modal data, gene expression data, and clinical information. On the METABRIC data set, the 5-year survival forecast accuracy of 82% was achieved. Meanwhile, Sun et al. [16] proposed a hybrid model, which can predict the survival time of breast cancer by combining I-RELIFE, a gene feature selection method, and support vector machine, using gene expression data and clinical information at the same time. In spite of the significant improvements achieved in these studies* via* combining multimodality data, it is still challenging with the fusion of multiple feature extraction methods to obtain better feature representation and consider the relationship between multimodality data.

Recently, the Attention method is proposed and it is argued that it is able to adaptively consider the importance of a single feature to the final global feature representation. It assigns different weights to each part of the feature sequence, extracting more critical and important information, allowing the model to make more accurate judgments. Based on this attribute, it has been widely used in machine translation and speech recognition. For example, Bahdanau et al. [17] proposed an encoder-decoder neural network based on Attention mechanism, which uses Attention mechanism to calculate the degree of association between each word in the input sequence and a particular word in the output sequence, so as to explain the corresponding relationship between French and English words. It not only achieves better translation effect, but also facilitates the calculation and storage of the model. Chorowski et al. [18] proposed an end-to-end trainable speech recognition model based on the Attention mechanism, which can combine the content and location information to select the next position in the input sequence for decoding. It is thus possible to identify speech inputs that are much longer in length than the training data. Google mind team [19] used the Attention mechanism to automatically capture local features of images in the field of computer vision to realize image classification. Similarly, the original data may contribute to the final representation differently. So we assume that the Attention mechanism can fully consider the importance within data and explore the correlation between multimodality data for better representation. To the best of our knowledge, there are no previous works which fuse features from different feature extraction algorithms with the help of Attention mechanism.

In this paper, we propose a deep neural network model (AMND) based on the Attention mechanism which fuses the patients gene expression data and clinical data for the breast cancer prognosis. The preprocessed gene expression profile data is decomposed by AMND with five algorithms, NMF_mu (NMF based on multiplicative update algorithms), NMF_als (NMF based on Alternating Least Square algorithms), NMF_alsobs (NMF based on Optimal Brain Surgery and Alternate Least Square algorithms), NMF_pg (NMF based on projection gradient algorithms), and PNMF (probabilistic nonnegative matrix factorization), respectively. Through the five algorithms, five characteristic matrices can be obtained. In order to individualize the importance of representations obtained from different NMF methods, Attention mechanism is introduced to calculate the weight of these representations of each sample according to its clinical data. After that, the weighted summating of these representations obtained by five single NMF methods is concatenated with clinical data, which serves as the final feature representation. This representation is input into the deep neural network for the classification task. This method not only takes into account the multimodality data, but also integrates multiple feature extraction methods, which can fully extract the high-level feature expression of gene expression data and clinical data, so as to improve the prognostic performance of breast cancer. Importantly, this method can be extended to survival prediction studies of other tumors, which provides a new strategy for other diseases prognosis.

## 2. Proposed Method

### 2.1. Feature Selection

#### 2.1.1. Nonnegative Matrix Factorization

Nonnegative matrix factorization (NMF) [20] refers to the search for nonnegative matrix *W*_*m*×*r*_ and *H*_*r*×*n*_ given a nonnegative matrix *V*_*m*×*n*_ and a positive integer *r*, (*r* ≪ min⁡{*m*, *n*}). It can be presented as follows:

(1) $$\begin{array}{c} V\approx WH \end{array}$$

where *r* is smaller than *m* or *n*, forcing the dimensions of *W* and *H* to be less than the dimensions of the original matrix. If *V*_*m*×*n*_ represents the pretreatment gene expression data matrix, *m* represents the number of samples, and *n* represents the number of genes, the NMF algorithm is to decompose the pretreatment genetic data matrix into feature matrix *W*_*m*×*r*_ and coefficient matrix *H*_*r*×*n*_, so as to achieve dimensionality reduction. In general, the objective function is used to guarantee the approximation effect before and after NMF factorization. Lee and Seung [21] gave two cost functions for judging convergence.

Cost function based on Euclidean distance squared is as follows:

(2) $$\begin{array}{c} {\left\|\;V-WH\;\right\|}^{2}=\underset{ij}{\sum }{\left(\;V_{ij}-{\left(\;WH\;\right)}_{ij}\;\right)}^{2} \end{array}$$

If and only if *V* = *WH*, (2) gives the optimal solution.

Cost function based on generalized KL (Kullback-Leibler) divergence is as follows:

(3) $$\begin{array}{c} D\left(\;V||WH\;\right)=\underset{ij}{\sum }\left(\;V_{ij}\log\frac{V_{ij}}{{\left(WH\right)}_{ij}}-V_{ij}+{\left(\;WH\;\right)}_{ij}\;\right) \end{array}$$

If and only if *V* = *WH*, (3) gets the minimum value.

The nonnegative matrix factorization problem is not only a nonconvex optimization problem, but also a NP hard problem [22]. Therefore, in order to find the optimal solution of W and H, various improved NMF algorithms are proposed.

#### 2.1.2. NMF Based on Multiplicative Update Algorithms

Lee and Seung [20] proposed NMF based on multiplicative update rules, which is simply noted as NMF_mu in this paper. It combines the two rules of gradient descent and multiplicative iteration skillfully and overcomes their respective disadvantages. The specific steps of the algorithm are as follows:

(a) Initialization matrix *W* ≥ 0 and matrix *H* ≥ 0.

(b) Iterate the matrix W and matrix H, respectively.

The updating rule of (2) is

(4) Wia←WiaVHTiaWHHTia

(5) Hau←HauWTVauWTWHau

The updating rule of (3) is

(6) Wia←Wia∑uHauViu/WHiu∑vHav

(7) Hau←Hau∑iWiaViu/WHiu∑kWka

(c) Repeat steps (b) until convergence occurs.

#### 2.1.3. NMF Based on Alternating Least Square Algorithms

Although the nonnegative matrix factorization is a nonconvex optimization problem, when the matrix W is fixed, it is a convex optimization problem for the matrix H, that is, the convex optimization problem of finding the optimal factor H for the fixed factor W. Paatero et al. [23] proposed NMF algorithm based on alternating least squares (ALS) algorithms, which is simply noted as NMF_als in this paper. The specific steps of the algorithm are as follows:

(a) Initialize matrix *W* ≥ 0.

(b) Fix matrix W and update H with formula (8):

(8) $$\begin{array}{c} H\leftarrow arg\underset{H\in R^{r\times n},H\geq 0}{min}{\left\|\;V-WH\;\right\|}_{F}^{2} \end{array}$$

(c) Fix matrix H and update W with formula (9):

(9) $$\begin{array}{c} W\leftarrow arg\underset{W\in R^{m\times r},W\geq 0}{min}{\left\|\;V-WH\;\right\|}_{F}^{2} \end{array}$$

Loop until convergence or maximum number of iterations is reached.

#### 2.1.4. NMF Based on Optimal Brain Surgery and Alternate Least Square Algorithms

Optimal Brain Surgery algorithm (OBS) [24, 25] is a network pruning algorithm based on Hessian matrix. The steps of the algorithm are as follows:

(a) Construct a local model of the error surface and analyze the influence of the weight disturbance. Taylor expansion of the error function is as follows:

(10) $$\begin{array}{c} \partial E={\left(\;\frac{\partial E}{\partial \omega }\;\right)}^{T}\partial \omega +\frac{1}{2}\delta \omega^{T}H\delta \omega +O\left(\;{\left\|\;\delta \omega \;\right\|}^{3}\;\right) \end{array}$$

where H is the Hessian matrix, T represents the transposition of the matrix, *ω* is the parameter in the neural network, and E is the training error of the training set. The pruning algorithm is applicable to any optimization algorithm.

(b) The constraint optimization problem can be solved by Lagrange multiplier method.

(11) $$\begin{array}{c} S=\frac{1}{2}△\omega^{T}H△\omega -\lambda \left(\;l_{i}^{T}△\omega +\omega_{i}\;\right) \end{array}$$

where *λ* is Lagrange multiplier. Using the inverse of the matrix, the optimal change in weight vector *ω* is obtained:

(12) $$\begin{array}{c} △\omega =-\frac{\omega_{i}}{{\left[H^{-1}\right]}_{i,i}}H^{-1}l_{i} \end{array}$$

(c) The corresponding optimal value of Lagrange operator S for element *ω*_*i*_ is

(13) $$\begin{array}{c} L_{i}=\frac{\omega_{i}^{2}}{2{\left[H^{-1}\right]}_{i,i}} \end{array}$$

where *H*^−1^ is the inverse of the Hessian matrix and [*H*^−1^]_*i*,*i*_ is the (*i*, *i*)*th* element in the inverse matrix. In the OBS process, the weight of the minimum eigenvalue will be deleted, and the remaining weight will be corrected according to (12).

NMF is based on Optimal Brain Surgery and Alternate Least Square algorithms, which is simply noted as NMF_ alsobs in this paper. NMF_alsobs is based on OBS algorithm to iteratively optimize W and H in (8) and (9). The optimization steps are as follows:

(a) Based on the iterative optimization problem of alternate least squares, a local model of the error surface is constructed to analyze the impact of negative perturbations in the matrix.

(b) Construct Lagrange operator to solve the constraint optimization problem.

(c) Get the optimal W or H.

#### 2.1.5. NMF Based on Projection Gradient Algorithms

Because of

(14) $$\begin{array}{c} F\left(\;W,H\;\right)=\frac{1}{2}{\left\|\;V-WH\;\right\|}_{F}^{2}=\frac{1}{2}\overset{n}{\underset{i=1}{\sum }}{\left\|\;V_{⋆i}-{\left(\;WH\;\right)}_{⋆i}\;\right\|}_{2}^{2} \end{array}$$

the nonnegative matrix factorization problem can be regarded as *n* independent nonlinear optimization problems on convex sets. The following nonlinear optimization problems can be solved using the projection gradient method:

(15) $$\begin{array}{c} \underset{x\in R_{+}^{n}}{min}f\left(\;x\;\right) \end{array}$$

where *f*(*x*) is the differentiable function defined on *R*^*n*^. Lin [26] proposed projection gradient methods for NMF, which is simply noted as NMF_pg in this paper and solve (15). The specific steps of the algorithm are as follows:

(a) Input: constant *β* and *σ*, where 0 < *β* < 1, 0 < *σ* < 1; initial feasible point *x*^1^.

(b) For the number of iterations, that is, *k* = 1,2, 3,4…,

(16) $$\begin{array}{c} x^{k+1}=P\left[\;x^{k}-\alpha_{k}▽f\left(\;x^{k}\;\right)\;\right] \end{array}$$

where *α*_*k*_ = *β*^*t*_*k*_^, *t*_*k*_ takes the values 1,2, 3… in turn. When *α*_*k*_ satisfies formula (17), the value of *t*_*k*_ is stopped, and it is denoted as *t* at the same time.

(17) $$\begin{array}{c} f\left(\;x^{k+1}\;\right)-f\left(\;x^{k}\;\right)\leq \sigma ▽f{\left(\;x^{k}\;\right)}^{T}\left(\;x^{k+1}-x^{k}\;\right) \end{array}$$

Check whether *x*^*k*+1^ satisfies the following convergence criterion:

(18) ▽pfxk≤ϵ▽fx1

(19) ▽pfxi≡=▽fxili≤xi≤uimin⁡0,▽fxixi=limax⁡0,▽fxixi=ui

When the conditions above are satisfied, then {*x*^*k*^}_*k*=1_^*∞*^ is output and the algorithm stops. If not, repeat step (b) until the conditions satisfied.

#### 2.1.6. Probabilistic Nonnegative Matrix Factorization

Since the gene expression profile data contains fixed noise, it is necessary to take the random characteristics of the data into account to conduct systematic processing and analysis. Belhassen Bayar et al. [27] proposed a probabilistic nonnegative matrix factorization algorithm, which is simply noted as PNMF in this paper. It extends the architecture and algorithm of NMF in random cases and assumes that the data is obtained from a polynomial probability density function.

The objective function of PNMF is

(20) $$\begin{array}{c} R\left(\;W,H\;\right)={\left\|\;V-WH\;\right\|}_{F}^{2}+\alpha {\left\|\;W\;\right\|}_{F}^{2}+\beta {\left\|\;H\;\right\|}_{F}^{2} \end{array}$$

The iteration rules are as follows:

(21) Wij←WijVHTijWHHT+αWij

(22) Hij←HijWTVijWTWH+βHij

Under the iterative rule of (21) and (22), the objective function of (20) is nonadditive, while the function R is fixed when W and H are fixed at a point.

#### 2.1.7. Nonnegative Double Singular Value Decomposition

The nonnegative matrix factorization algorithm is a nonconvex optimization process in the iteration. Furthermore, the result of the iteration depends to some extent on the initial value, which is generated randomly. As a result, the selection of the initial values of W and H will directly affect the iterative results of the decomposition algorithm. Most NMF algorithms in the literature use random nonnegative initialization for (W, H). Iterates converge to a local minimum, so it becomes necessary to run several instances of the algorithm using different random initializations and then select the best solution. This obviously reduces the efficiency and real-time performance of the algorithm. Therefore, we use the method of Nonnegative Double Singular Value Decomposition [28] as an initialization strategy, which makes the NMF model converge more quickly within a limited number of iteration steps and can be combined with all available NMF algorithms readily. The following is a detailed description of the initialization process:

Let the singular value decomposition of matrix Y be

(23) $$\begin{array}{c} Y=M\Sigma N^{T} \end{array}$$

From the singular value decomposition of matrix Y, it can be seen that the matrix *Y*_*k*_ consists of the largest K pairs of singular values (*σ*_*i*_, *m*_*i*_, *n*_*i*_)_*i*=1_^*k*^ of matrix Y,

(24) $$\begin{array}{c} Y_{k}=\overset{k}{\underset{i=1}{\sum }}\sigma_{i}m_{i}n_{i}^{T} \end{array}$$

which is the best 2 norm approximation of the matrix Y with rank k (k≤ rank(Y)). If the matrix Y is a nonnegative matrix, it can be seen from Perron-Frobenius theorem that *m*_1_ and *n*_1_ are also nonnegative vectors, so the first column of the matrix W can be

(25) $$\begin{array}{c} W\left(\;:\text{,}1\;\right)=\sqrt{\sigma_{i}}m_{1} \end{array}$$

Similarly, the first row of matrix H can be

(26) $$\begin{array}{c} H\left(\;1,\text{:}\;\right)=\sqrt{\sigma_{i}}n_{1}^{T} \end{array}$$

The following singular vectors *m*_2_ and *n*_2_ may contain negative elements due to the orthogonality of singular value decomposition. For the matrix *X* = *σ*_2_*m*_2_*n*_2_, the negative element in X is replaced by 0, and the remaining elements are unchanged, that is, taking the positive part *X*_+_ of matrix Y. As an approximate matrix of X, *X*_+_ is subjected to singular value decomposition, and W is initialized by the first singular value vector of *X*_+_. The initialization of other columns is the same. In this way, NNDSVD initial matrix is constructed as the initial value of nonnegative matrix.

### 2.2. Attention-Based Multi-NMF Deep Neural Network

The NMF algorithm decomposes the nonnegative matrix into two matrixes in multiplication forms without changing the original data structure. Because there is no negative value in the process of NMF and the factorization results are highly interpretable, NMF analysis of gene expression data makes the research results more valuable [29]. Compared with the traditional methods, the NMF algorithm is not only simple to implement, but also takes up very little storage space. As a result, NMF has a wide range of applications in the field of biological information. The NMF_mu algorithm combines the two rules of gradient descent and multiplicative iteration skillfully and overcomes their respective shortcomings. However, in practical application, neither local convergence is guaranteed nor stable performance is obtained. At the same time, 0 deadlock will also occur during iteration. In order to ensure the convergence of the algorithm, NMF_als can be used to optimize the loss function of nonnegative matrix factorization. Each iteration of the algorithm will reduce the error, so the result will definitely converge. On the basis of NMF_alsobs, the error is reduced by removing the weight of the minimum eigenvalue, so as to accurately solve W and H. NMF_pg is one of the classical methods for solving boundary constraint optimization problems, whose advantage is that the convergence is easy to guarantee and only gradient information is used to judge each iteration. NMF_pg has better convergence than NMF_mu and can effectively avoid the 0 deadlock phenomenon encountered by NMF_mu. However, NMF_pg converges slowly. The PNMF algorithm avoids the errors and noises generated during the measurement or observation of gene expression profile data. The feature vectors decomposed by different NMF improved algorithms are different. Usually, the eigenvalue matrices can only reach the local optimal solution, and important features in the original data cannot be completely expressed. Simply using a single NMF algorithm can even lose some important genetic features. Therefore, in order to better express the characteristics of the original gene expression profile, this paper proposes an AMND model, which combines the feature vectors decomposed by the above five NMF algorithms through Attention mechanism. It can not only compensate for the loss of important information, but also obtain better feature representation.

Attention mechanism was originally used to deal with alignment problems in machine translation, because each word in the original sentence may contribute to a word in the target sentence with different contribution. Attention mechanism, however, can adaptively consider the importance of each word in the original sentence to the word in the target sentence. Similarly, the eigenvectors obtained by different NMF algorithms are only an approximation of the original data matrix and can not fully represent the information of the original data. Therefore, the concept of Attention mechanism applied in RNN is used to generate a more biological feature vector by adaptively summing the eigenvectors obtained by different NMF algorithms. In this way, not only was the correlation between clinical data and gene expression data considered, but also the feature vectors obtained by multiple NMF improved algorithm were combined to better express the original data and further improve the prediction performance.

**Figure 1:**
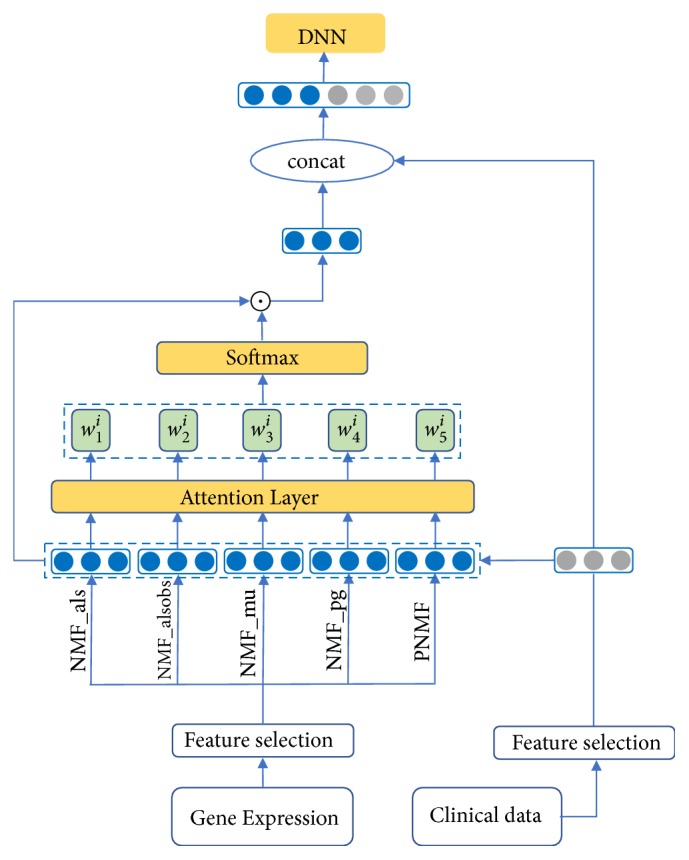
The overall process of our AMND model for the breast cancer prognosis prediction.

This paper focuses on the efficient fusion of multiple feature extraction algorithms. The commonly used method is to directly concatenate the results obtained by multiple feature extraction methods or to carry out weighted summation. However, direct weighted summation results in the same weight for each feature extraction method, which is not the most effective. Therefore, we propose the AMND method to solve this problem. The model effectively fuses Multi-NMF to obtain new gene expression feature vectors, which is fused with clinical data features and put into DNN for prediction. The structure of the AMND is shown in Figure 1. First, we use NMF_mu, NMF_als, NMF_alsobs, NMF_pg and PNMF algorithms to extract the features of gene expression profile data and obtain five feature matrices. Then, the weighted sum of each NMF improved algorithms is obtained by the Attention mechanism. Different from direct weighted summation, Attention mechanism calculates the weight of each NMF improved algorithm adaptively, according to the clinical data of each patient sample. *F*_*j*__*x*_*i*_ is used to represent the eigenvector of the i-th sample in the eigenvalue matrix obtained by the j-th NMF decomposition method, and the clinical feature vector of the sample is denoted as *C*_*x*_*i*_. So, Weight calculation formula is as follows:

(27) $$\begin{array}{c} w_{j}^{i}={\left(\;F_{j}\_x_{i}\;\right)}^{T}X\left(\;C\_x_{i}\;\right) \end{array}$$

where T represents transposition and X is a weight matrix used to establish a relational mapping between *F*_*j*__*x*_*i*_ and *C*_*x*_*i*_. *w*_*j*_^*i*^ is the weight corresponding to the feature vector of the i-th sample in the j-th NMF decomposition algorithm. According to formula (27), *F*_*j*__*x*_*i*_ and the *C*_*x*_*i*_ are input into neural network which can get *w*_*j*_^*i*^. The weights obtained are normalized using* softmax* function. For example, the normalized $\hat{w_{j}^{i}}$ can be considered as the contribution of j-th NMF algorithm to the i-th sample.

(28) $$\begin{array}{c} \hat{w_{j}^{i}}=\frac{e^{w_{j}^{i}}}{\sum_{j=1}^{5}e^{w_{j}^{i}}} \end{array}$$

Finally, the weighted sum of the Multi-NMF is used to obtain F:

(29) $$\begin{array}{c} F=\overset{5}{\underset{j=1}{\sum }}\hat{w_{j}^{i}}F_{j}\_x_{i} \end{array}$$

However, F obtained from the above equation actually contains only gene expression profile data. In order to consider the multimodality data, F is fused with clinical data and put into DNN for classification prediction. AMND is an end-to-end model where DNN parameters can be optimized and adjusted through training.

## 3. Results and Discussion

### 3.1. The Data

We downloaded the METABRIC breast cancer dataset from the website Synapse* (synapse.sagebase.org)* and used the dataset's gene expression and clinical data. The METABRIC dataset used in this study included 1980 samples. Moreover, according to the research work of Khademi et al. [30], five-year slot was used as the threshold to classify the two types of patients. Among them, 491 patients were divided into short-term survival samples and 1,489 patients into long-term survival samples. Meanwhile, the labels of short survival samples were set to 0 and the long life samples to 1. For gene expression data, handling methods of Sun et al. [31] were used to preprocess it. Then, the processed gene expression profile data and clinical data are normalized to between 0 and 1. For gene expression profile data, five NMF algorithms are used to extract the features of the same dimension, especially with the feature dimension of 200. Each sample contained 25 dimensions of clinical information, such as age of diagnosis, tumor size, cancer grade, etc. In order to evaluate the performance of the algorithm, in this paper we randomly divide the data set into three groups, that is, 80% of the samples do training set, 10% of the samples do test set, and the remaining 10% of the samples do verification set. The division of data sets can be seen from Table 1. The training set is used to train the model, while the verification set is used to adjust the parameters of the neural network model, and the test set is used to test. The experimental results in this paper are all from the test set.

### 3.2. Experimental Results

For the performance evaluation of AMND, we plot the ROC curve to show the interaction between True Positive (TP) and False Positive (FP) by changing the threshold, and calculate the AUC. In addition to AUC, Accuracy (Acc), Precision (Pre), F1-score, and recall are also used for performance evaluation. The following experimental results are derived from the average of the results obtained from 100 repartitioning data sets.

#### 3.2.1. Compare with the Model of Single NMF Improved Algorithm

**Figure 2:**
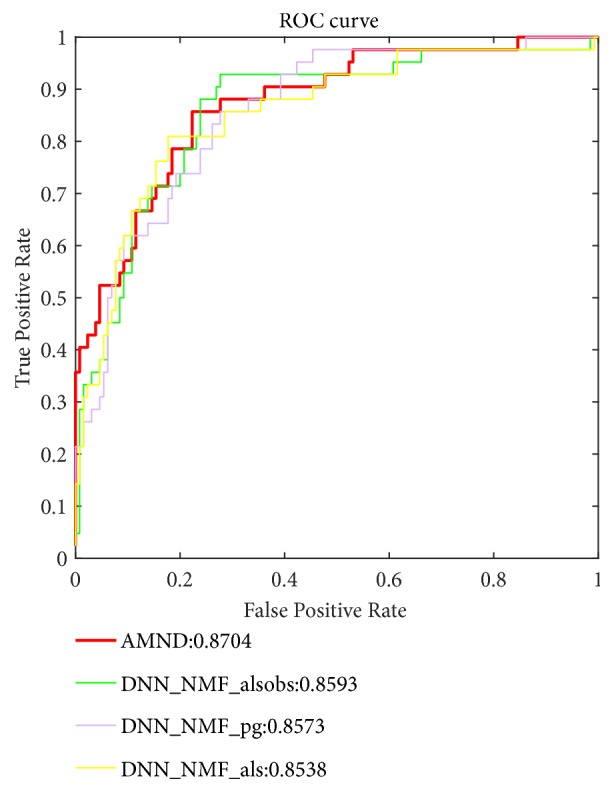
ROC curves obtained using the AMND and Multi-NMF.

**Figure 3:**
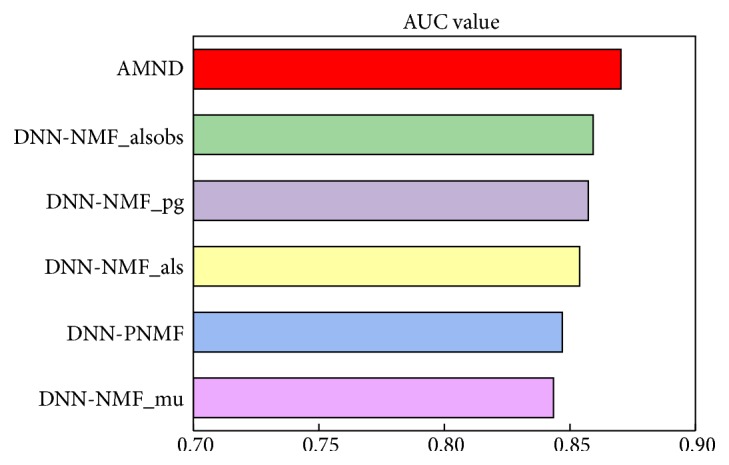
The AUC values of AMND and Multi-NMF.

In order to verify the effectiveness of Multi-NMF algorithm fused by Attention mechanism, we compare the results of our model with that of algorithms using single NMF algorithm on the dataset. Here, we choose the model of the three most effective single NMF improved algorithm to draw ROC curve together with AMND. As shown in Figure 2, compared with the model based on a single NMF improved algorithm, AMND has better overall performance. In addition to the ROC curve, the corresponding AUC values of each method are also calculated, as shown in Figure 3. Compared with the deep neural network models using other five NMF improved algorithms, namely, DNN-NMF_mu, DNN-NMF_PNMF, DNN-NMF_als, DNN-NMF_pg, and DNN-NMF_alsobs, AMND obtained the best AUC value (87.04%). The predictive performance of AMND was improved by 2.34%, 2.69%, 1.66%, 1.31%, and 1.11%, respectively, compared with the deep neural network model based using single NMF improved algorithm.

**Figure 4:**
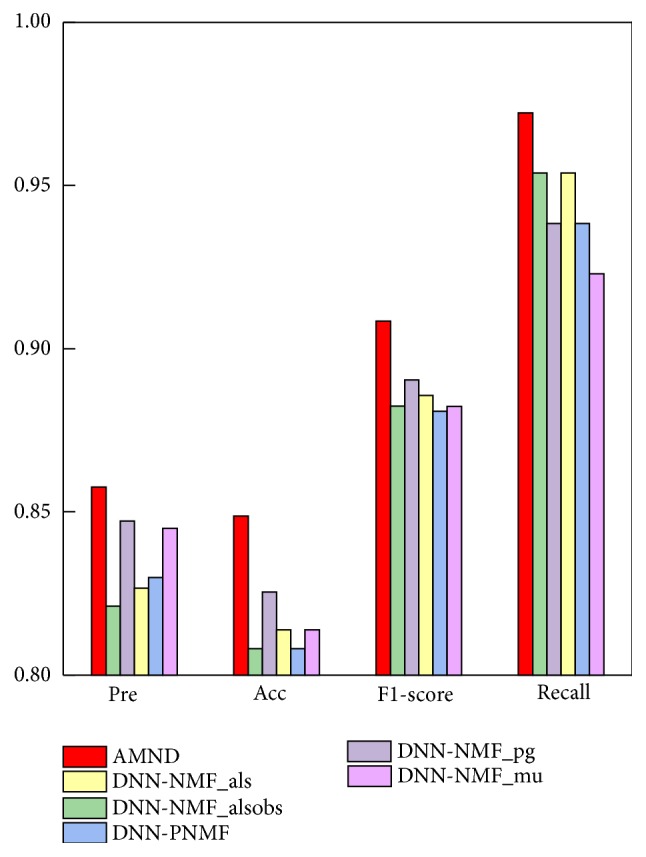
Predicted performance of AMND and Mutli-NMF on Acc, Pre, F1-score, and recall.

In addition, the Acc, Pre, F1-score, and recall performance indicators of the five deep neural network models using single NMF improved algorithm were compared with that of AMND, and the results were shown in Figure 4. The results show that the overall performance of AMND is better than that of the other five models using single NMF improved algorithm. Meanwhile, the Acc, Pre, F1-score, and recall values corresponding to the six methods are shown in Table 2. The Acc-value of DNN-NMF_PNMF, DNN-NMF_als, and DNN-NMF_pg were 80.81%, 81.39%, and 82.55%. AMND obtained the highest prevalue, 84.88%, which is 4.07%, 3.49%, and 2.33% higher than that of DNN-NMF_PNMF, DNN-NMF_als, and DNN-NMF_pg, respectively. The results showed that the forecast level of AMND for both positive and negative samples was better than the other five models. In addition, in terms of Pre indicators, AMND also achieved corresponding improvement, with a 1.65% increase in AMND over DNN-NMF_alsobs. All the above comparison results show that the overall performance of AMND is better than that of the model using single NMF improved algorithm. It can be concluded that using single NMF improved algorithm does lose some important information with in the original data. Furthermore, the fusion technology of multiple feature extraction algorithms based on Attention mechanism play a significant role in compensating for that loss of information and improving the performance of cancer prediction.

#### 3.2.2. Compare with Variants of the Proposed Model

In order to verify the effectiveness of Attention mechanism in AMND model and the significance of fusing multimodality data, we designed the following four comparative experiments:

- Clinical data are only used for weight calculation of Attention mechanism
- In this experiment, clinical data only provide supervised information for computing the weights of Attention mechanism, and the eigenvectors obtained by the five NMF algorithms are weighted and summed using the obtained weights. The final eigenvectors obtained by weighted summation are directly input into the neural network for classification. The purpose of this experiment is mainly to verify the effectiveness of the Attention mechanism. The corresponding model is named clinical_first here.
- Clinical data are only used to fuse multimodality data
- In this experiment, the eigenvectors obtained by the five NMF algorithms are respectively assigned with weights of 0.2 and summed (assuming each contributes equally to the representation). Then this middle representation is concatenated with clinical data to obtain the final representation, which is then put into neural network for classification. In this variant, the Attention mechanism is not used and this variant is named clinical_second here.
- Neural network model based on clinical data
- Many clinical features are directly related to prognosis. Therefore, clinical data are directly input into the neural network for training and prediction to verify the validity of the fusion of multimodality data. The corresponding model is named only_clinical here.
- Neural network model based on gene expression profile data
- In this experiment, the eigenvectors obtained by the five NMF algorithms are, respectively, assigned with weights of 0.2, and the final eigenvectors obtained by weighted summation are input into the neural network for classification. The corresponding model is named only_exp here.

**Figure 5:**
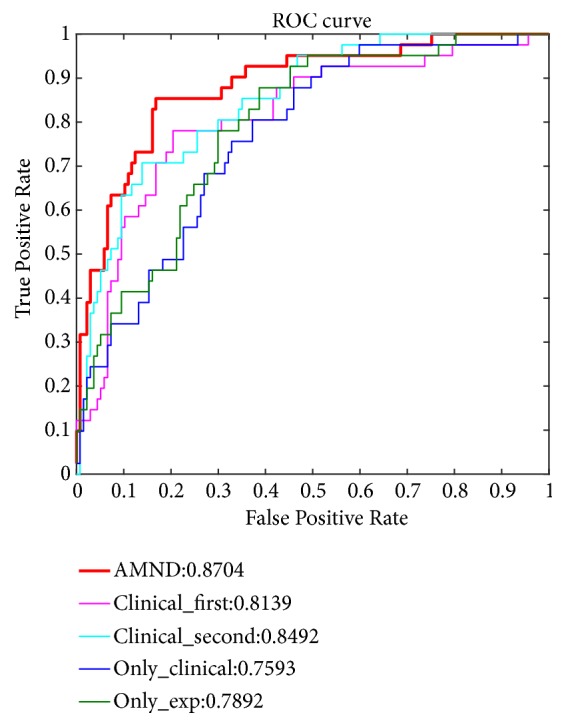
1ROC curves of AMND algorithm, clinical_first, clinical_second, only_clinical, and only_exp models.

The experimental results are shown in Figure 5. From the figure, we can draw the following conclusions: AMND achieves the best results. Its AUC value reaches 87.04%, which is higher than clinical_second, clinical_first, only_clinical, and only_exp by 2.12%, 5.95%, 11.11%, and 8.12%, respectively. The results of clinical_first and clinical_second show that the good effect of AMND is closely related to the two uses of clinical data. The first is to calculate the weight by Attention mechanism, and the second is to fuse multimodality data. That is to say, Attention mechanism and fusion of multimodal data both can improve the predictive performance of breast cancer survival. The results of clinical_first and only_exp show that the eigenvectors obtained by weighted summation of five NMF algorithms using Attention mechanism are more representative than those obtained by weighted summation based on the same weight. It proves that Attention mechanism, an adaptive method of calculating weights, can better fuse the eigenvectors obtained by five NMF algorithms and thus get better feature representation. From only_clinical and only_exp, we can see that clinical data do have a direct impact on the prognosis, but the effect is not obvious. Therefore, the feature representation obtained by fusing multimodality data is more representative and contains more biometric information.

#### 3.2.3. Performance Comparison of Existing Methods

**Figure 6:**
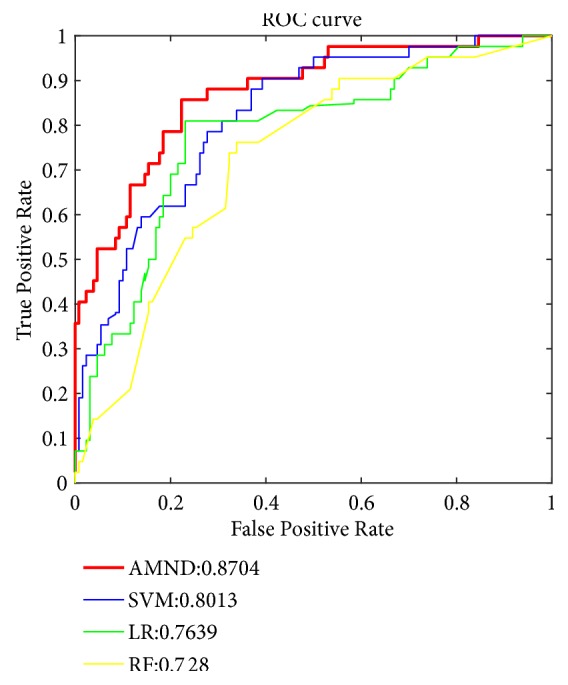
ROC curves of AMND algorithm and SVM, LR, and RF methods.

**Figure 7:**
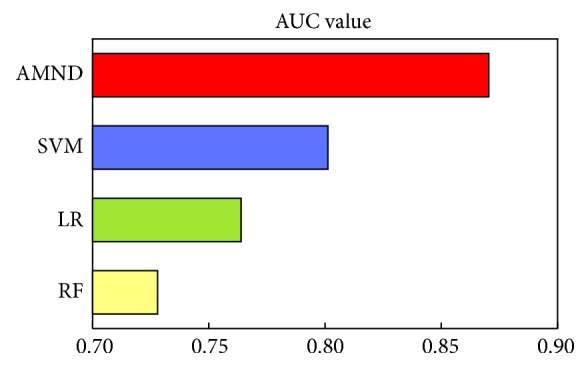
AUC values of AMND algorithm and SVM, LR, and RF methods.

In order to further verify the good effect of the proposed method on the prediction of breast cancer survival, this paper also compared AMND with SVM, LR, and RF.  Figure 6 shows the ROC curve of the four methods. It can be seen from the figure that the overall performance of the AMND method is better than other methods. In addition to the ROC curve, the corresponding AUC value of each method is also calculated, as shown in Figure 7. The AUC values of SVM, LR, and RF were 80.13%, 76.391%, and 72.8%, respectively. The AUC value of the AMND method is 87.04%, which is 6.91%, 10.65%, and 14.24% higher than that of the other three methods. These results indicated that the fusion of multimodality data was significantly helpful to improve the predictive performance of breast cancer survival, and the AMND method could better use multiple feature extraction methods to improve the prediction accuracy of survival.

**Figure 8:**
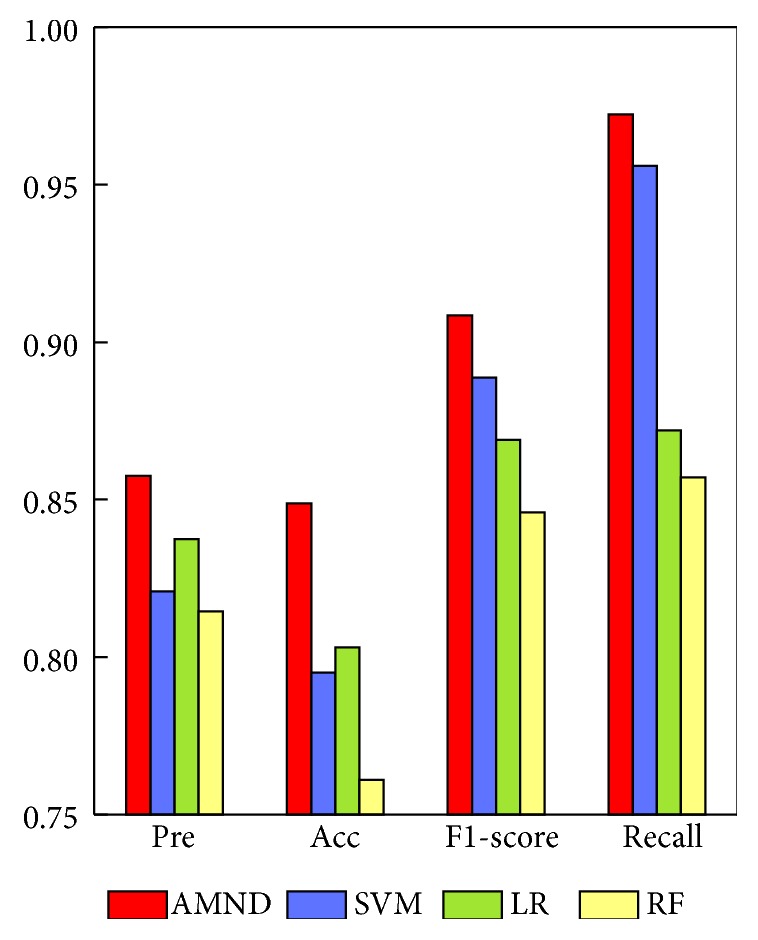
Prediction performance of AMND algorithm and SVM, LR, and RF methods.

This paper also analyzes the values of Acc, Pre, F1-score, and recall of different methods. The corresponding results are shown in Figure 8 and Table 3. As demonstrated in Figure 8, the performance of AMND method on Acc, Pre, F1-score, and recall is higher than the other three methods. AMND is higher than SVM methods on Acc, Pre, F1-score, and recall by 5.37%, 3.68%, 1.96%, and 1.63%, respectively. In addition, compared with LR and RF, AMND also achieved better performance. In summary, AMND is superior to other methods under different performance evaluation indexes, indicating that it performs well when making the prediction of breast cancer survival.

In order to verify the performance of the model, it is compared with the results obtained from similar studies. Sun et al. [31] conducted a survival prediction study on gene expression profile, CAN spectrum and clinical data in METABRIC data and proposed MDNNMD method. The AUC value obtained in their study was 84.5%. Gevaert et al. [15] proposed a predictive algorithm based on Bayesian network, which is noted as BPIM. This algorithm fully integrated the two kinds of modal data, namely, gene expression data and clinical information. They obtained the predictive performance of 84.5% AUC value in the prediction of breast cancer survival. Khademi et al. [30] proposed a probabilistic graph model (PGM) incorporating gene expression profiling and clinical data from METABRIC data and obtained an AUC value of 82%. As shown in Table 4, the AUC values of AMND were 2.54%, 2.54%, and 5.04% higher than that of MDNNMD, PGM, and BPIM, respectively. Thus, AMND has achieved good results in predicting the survival of breast cancer.

In conclusion, the AMND model proposed in this paper improves the prediction accuracy of breast cancer prognosis prediction research. It can not only help patients understand their life expectancy, but also provide a theoretical support for clinicians in making medical decisions and avoid wasting medical resources. Firstly, NMF algorithm is used to extract features from the original gene expression profile data, which can be high-dimensional and hard to be directly used. Therefore, using NMF algorithm can reduce the dimension of the gene data. From a biological point of view, each line in matrix W obtained by NMF can be regarded as a combination of the different features within the original gene data for each sample. Therefore, the decomposed W is the characteristic matrix and H is the coefficient matrix. W not only reduces the dimension based on the original gene matrix, but also achieves the purpose of feature extraction. Secondly, based on five NMF decomposition algorithms, five low-dimensioned eigenvectors are obtained, which are then fused by Attention mechanism to generate a more biologically meaningful feature representation, which can greatly help the downstream classification task. Finally, we use multimodality data and deep learning methods in our proposed model. Not only can better low-dimensional representation of the original data be obtained, but also higher classification performance can be achieved.

## 4. Conclusions

In a summary, a deep neural network model based on Attention mechanism (AMND) was proposed for prediction of breast cancer. To effectively extract useful information within the gene profile data, clinical data is first used to compute the weights of five eigenvectors obtained by five NMF algorithms. Then the weighted summation of five eigenvectors is concatenated with clinical data to generate the final representation, which is put into deep neural networks for classification. The AMND method is a preliminary attempt to study the prediction of the prognosis of breast cancer by the Attention mechanism. The results show that the use of the Attention mechanism can better consider the connection between patients' clinical data and gene expression data; furthermore, the results also demonstrate that the use of multimodality data can improve the representative ability of the final feature vector. We also compare our performance with an existing method, namely, MDNNMN. Our results show that the proposed model is superior to MDNNMN on multiple evaluation indexes. The most important success of this work is the improvements for the in-depth understanding breast cancer omics data and the development of relevant prediction methods for survival. Moreover, this method can be extended to predict the survival time of other cancer diseases, providing a new strategy for cancer prognosis.

## Figures and Tables

**Table 1:** Division of data of MITTABRIC dataset.

	Long time survivors	Short time survivors	Total
Training set	1191	393	1584
Testing set	149	49	198
Validation set	149	49	198
Total	1489	491	1980

**Table 2:** ACC, Pre, F1-score, and Recall predictive performance indexes of AMND and Mutli-NMF.

Method	Acc	Pre	F1-score	Recall
*AMND *	* 0.8488 *	* 0.8576 *	* 0.9084 *	* 0.9723*
DNN-NMF_alsobs	0.8081	0.8211	0.8825	0.9538
DNN-NMF_pg	0.8255	0.8472	0.8905	0.9384
DNN-NMF_als	0.8139	0.8266	0.8857	0.9538
DNN-NMF_PNMF	0.8081	0.8299	0.8808	0.9384
DNN-NMF_mu	0.8139	0.845	0.8823	0.923

**Table 3:** Comparison of Pre, Acc, F1-score, and recall between AMND, SVM, RF, and LR.

	Acc	Pre	F1-score	Recall
*AMND *	*0.8488 *	* 0.8576 *	* 0.9084 *	* 0.9723*
SVM	0.7951	0.8208	0.8888	0.9560
LR	0.8031	0.8375	0.8690	0.8720
RF	0.7610	0.8145	0.8460	0.8570

**Table 4:** Comparison of AMND and existing research results.

Method	AUC
*AMND *	* 87.04*%
MDNNMD	84.5%
BPIM	84.5%
PGM	82%

## Data Availability

The data used to support the findings of this study are available from the corresponding author upon request.
